# Vitamin D and cancer-related fatigue in elderly patients: mechanisms and therapeutic insights—a narrative review

**DOI:** 10.3389/fnut.2025.1642166

**Published:** 2025-09-29

**Authors:** Juan Wu, Jinzheng Chi, Yu Zhang

**Affiliations:** ^1^The Second Clinical Medical College of Zhejiang Chinese Medical University, Hangzhou, China; ^2^Department of Gastroenterology, Pei County People's Hospital, Xuzhou, China; ^3^Department of Medical Oncology, Zhejiang Hospital, Hangzhou, China

**Keywords:** cancer-related fatigue, elderly cancer patients, vitamin D, inflammation, muscle atrophy, personalized therapy

## Abstract

Cancer-related fatigue (CRF) is a prevalent and debilitating symptom in elderly cancer patients. According to the National Comprehensive Cancer Network (NCCN) and international consensus, CRF is defined as a persistent, multidimensional fatigue disproportionate to activity, unrelieved by rest, and involving physical, emotional, and cognitive domains. Diagnosis requires standardized patient-reported scales, objective biomarkers (e.g., inflammatory and metabolic indices), and exclusion of comorbidities such as anemia or organ dysfunction. In elderly patients, CRF arises from interrelated alterations, including chronic inflammation, neuroendocrine dysregulation, circadian disruption, and progressive muscle atrophy, that perpetuate a vicious cycle. Current treatments encompass pharmacological agents (e.g., corticosteroids, psychostimulants, antidepressants, and traditional Chinese medicine, primarily studied in China) and non-pharmacological modalities (e.g., exercise, acupuncture, and cognitive-behavioral therapy), yet efficacy remains inconsistent. Emerging approaches such as mitochondrial modulators and bright light therapy are expanding the therapeutic landscape. Vitamin D, particularly cholecalciferol (vitamin D_3_), is commonly deficient in older adults and shows promise in alleviating CRF through anti-inflammatory, immunomodulatory, neuroprotective, and myogenic effects. This narrative review summarizes current evidence on vitamin D_3_’s mechanisms and clinical value, highlights its role as a multi-target modulator, and explores its integration into personalized CRF management. Future studies should refine dosing strategies, clarify responses in the elderly, and assess the synergy between conventional and novel interventions.

## Highlights

1. This narrative review is the first to comprehensively summarize the potential mechanisms by which VD may alleviate CRF in elderly patients.
2. VD modulates immune responses that help relieve central fatigue.
3. VD reduces muscle fatigue through its anti-inflammatory effects.
4. VD is closely associated with improvements in mood and sleep.
5. This study provides new therapeutic targets and theoretical support for the management of CRF.

## 1 Introduction: the challenge of elderly cancer-related fatigue (CRF) and a new perspective on vitamin D (VD) intervention

CRF is one of the most common and debilitating symptoms in elderly cancer patients and survivors, with its prevalence rising alongside advances in cancer therapy. According to the National Comprehensive Cancer Network (NCCN), CRF is defined as a persistent, subjective sense of tiredness or lack of energy related to cancer or its treatment, disproportionate to recent activity, not relieved by rest, and interfering with daily functioning (1). Epidemiological studies report that up to 70% of older patients experience moderate to severe fatigue during diagnosis or treatment (2). CRF in the elderly is frequently accompanied by cognitive, sleep, and emotional disturbances, which further impair daily functioning and reduce quality of life (3). Moreover, CRF may compromise treatment tolerance and safety, negatively impacting prognosis (4). Mitochondrial dysfunction (5) and chronic low-grade inflammation (6) have been identified as key mechanisms driving CRF in the elderly.

VD deficiency is highly prevalent among older adults (≥ 65 years) and is recognized for its roles in muscle metabolism, immune regulation, and neuroprotection, all of which may be relevant to CRF pathophysiology (7). The VICTORIA randomized trial is currently investigating whether individualized VD supplementation can alleviate fatigue and improve quality of life in cancer patients (8). In elderly cancer populations, VD supplementation has demonstrated good safety and adherence, with daily doses of 4,000 IU recommended for CRF management (9).

In summary, CRF is a highly prevalent condition that substantially impairs quality of life and prognosis in elderly cancer patients. Given VD’s critical roles in multiple physiological pathways and its widespread deficiency in this population, exploring VD supplementation as a safe and effective strategy for CRF management has significant clinical and translational value. This review summarizes the mechanisms and interventions of CRF, with a focus on the biological pathways and clinical evidence supporting VD in improving fatigue, thereby providing a theoretical basis for precision nutrition strategies.

## 2 Multidimensional pathophysiological mechanisms of elderly CRF

**Figure 1:**
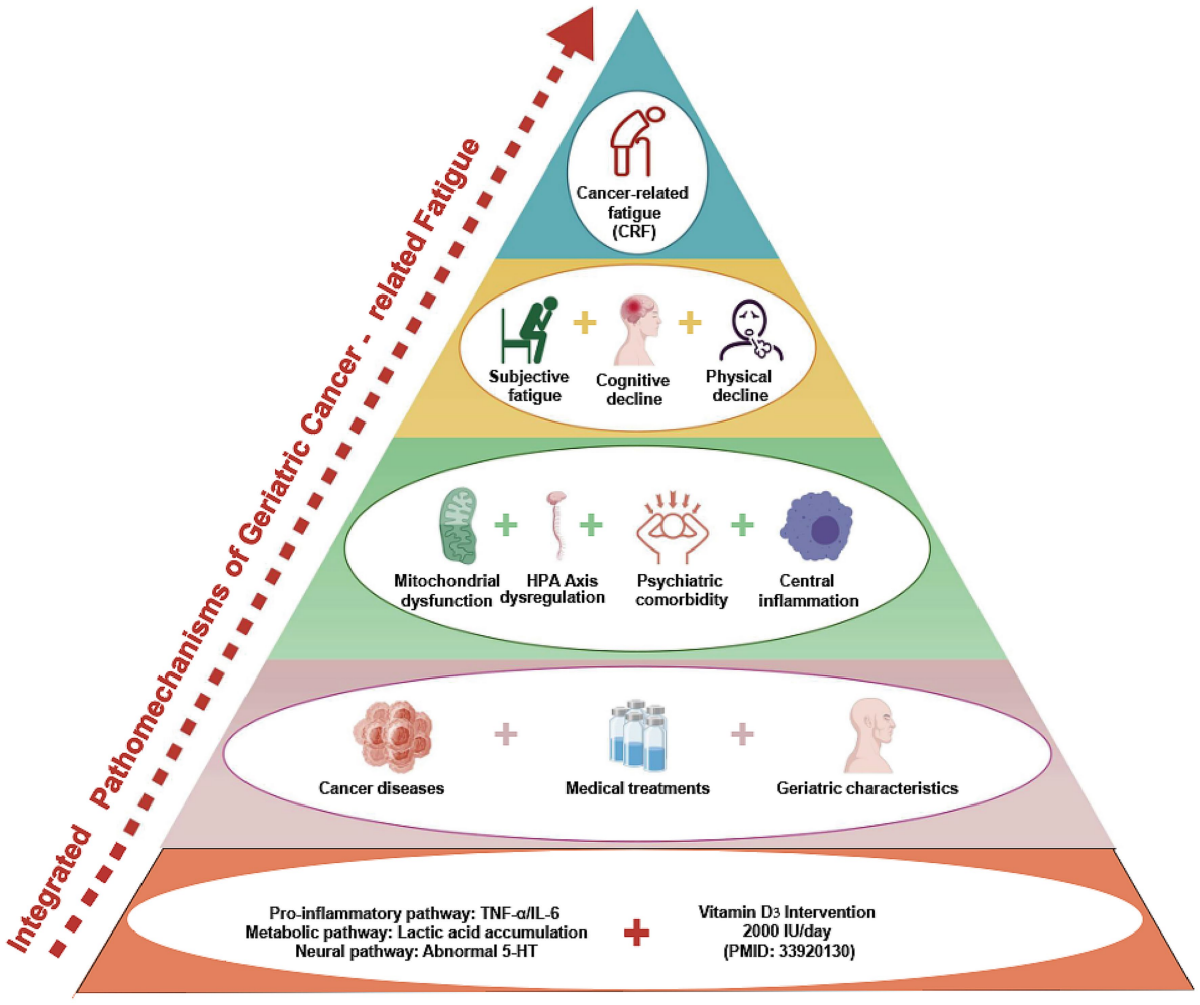
An integrated model of multiple mechanisms underlying elderly CRF.

CRF is a highly complex, multidimensional symptom resulting from the interaction of biological, psychological, and treatment-related factors. In the elderly population, overall functional decline exacerbates the progression and persistence of CRF. Although widely recognized as a core clinical problem, CRF currently lacks a unified definition and diagnostic standard (10). Clinical evaluation primarily relies on patient-reported outcome measures, such as the Brief Fatigue Inventory (BFI), the Functional Assessment of Chronic Illness Therapy-Fatigue (FACIT-F), and the Multidimensional Fatigue Inventory (MFI) (11). Numerous studies have examined its underlying mechanisms (4, 12), which can be broadly categorized into three domains: biological, psychological, and treatment-related mechanisms (Figure 1).

### 2.1 Biological mechanisms involving both central and peripheral impairments

In elderly patients, CRF shares similar underlying mechanisms with the general population and is commonly explained by central and peripheral fatigue hypotheses. Central fatigue refers to the impaired initiation or maintenance of voluntary activities due to abnormalities in central nervous system signaling, characterized by dysregulated inflammatory cytokines, hypothalamic–pituitary–adrenal (HPA) axis dysfunction, disruption of the circadian rhythm, and aberrant neuromodulation of serotonin (5-HT) and vagal pathways. Peripheral fatigue originates in the neuromuscular junction and skeletal muscle system, reflecting reduced responsiveness of peripheral neuromuscular units to central stimuli. This is primarily driven by impaired adenosine triphosphate (ATP) production or utilization, accumulation of metabolic by-products, and disrupted neuromuscular coupling (13–15). Beyond these biological mechanisms, psychosocial factors (e.g., depression, anxiety, social isolation) and treatment-induced mechanisms (e.g., chemotherapy-, radiotherapy-, or targeted therapy-related toxicity) also contribute significantly to the onset and persistence of CRF in the elderly.

#### 2.1.1 Central fatigue

Inflammation plays a central role in the development of central fatigue. Cancer itself or its treatments can activate pro-inflammatory cytokine networks, including C-reactive protein (CRP), interleukin-1 (IL-1), interleukin-6 (IL-6), interferon (IFN), and tumor necrosis factor-*α* (TNF-α). These mediators disrupt immune-neural signaling pathways and may induce fatigue-related responses in the central nervous system (16–18). Pro-inflammatory cytokines can also hyperactivate the hypothalamic–pituitary–adrenal (HPA) axis, leading to flattened or elevated nocturnal cortisol rhythms, impaired anti-inflammatory capacity, and exacerbated fatigue (19–21).

Recently, the vagus nerve has attracted growing attention in CRF. As the core of the “neuroimmune reflex arc,” it suppresses the release of TNF-*α* and IL-6 via acetylcholine-mediated anti-inflammatory pathways, and indirectly regulates HPA axis activity (22). Reduced vagal activity in the elderly is thought to amplify inflammatory responses and weaken neuroendocrine adaptation. Vagus nerve stimulation (VNS), an emerging neuromodulation technique, has demonstrated unique potential for CRF management by activating the “cholinergic anti-inflammatory pathway” and modulating the immune-neuro-endocrine network (23). The therapeutic effects of VNS are mainly attributed to three mechanisms: (1) activation of the cholinergic anti-inflammatory pathway, inhibition of NF-κB signaling, and reduction of systemic inflammation (24); (2) upregulation of brain-derived neurotrophic factor (BDNF), promotion of neurogenesis and synaptic plasticity, and repair of chemotherapy- or tumor microenvironment-induced neuronal damage (25); and (3) suppression of excessive hypothalamic corticotropin-releasing hormone (CRH) secretion, thereby restoring circadian cortisol rhythms (26).

In addition, dysregulation of the 5-HT system also contributes to CRF. Through its receptors, 5-HT modulates feeding, arousal, motor activity, and mood. CRF can be driven by synaptic accumulation of 5-HT (27), genetic susceptibility such as the 5-HTTLPR SS genotype (28), and impaired HPA axis feedback inhibition (29). Animal studies further support this mechanism, as 5-HT agonists induced delayed awakening, reduced cortisol, and influenza-like fatigue symptoms in mice (30), a phenomenon also observed in exercise-induced fatigue (31).

In summary, inflammation serves as the key driver of central CRF. The HPA axis, vagus nerve, and 5-HT system interact as an integrated network that underlies the neurobiological basis of CRF in the elderly, highlighting potential targets for therapeutic intervention.

#### 2.1.2 Peripheral fatigue

In elderly cancer patients, reduced nutritional intake, decreased metabolic rate, and insufficient muscle reserves often result in pronounced peripheral fatigue. Anemia and malnutrition can lead to decreased ATP synthesis, impairing muscle energy supply. At the same time, tumor cells produce large amounts of metabolic byproducts such as lactate through anaerobic metabolism. The accumulation of these substances in the body can impair muscle function and exacerbate symptoms of fatigue (32). A study by Sundberg et al. found that elderly individuals are more prone to muscle fatigue than younger individuals. *In vitro* fatigue simulation experiments further confirmed that this age-related decline in muscle function is primarily attributed to impaired excitation-contraction coupling and reduced efficiency of myosin-actin cross-bridge cycling (33). Therefore, elderly cancer patients should be considered a key population for the occurrence and management of peripheral fatigue.

### 2.2 Psychosocial mechanisms

Psychological factors are recognized as critical drivers of CRF in elderly cancer patients. Under stressors such as disease diagnosis, treatment-related burdens, and functional decline, patients frequently experience anxiety, depression, and helplessness, which can amplify fatigue through neuroimmune pathways. Symptom cluster studies indicate that fatigue commonly co-occurs with pain, sleep disturbances, loss of appetite, and memory impairment, suggesting overlapping central regulatory mechanisms such as serotonergic imbalance, disrupted cortisol rhythms, and prefrontal cortical inhibition (34).

Among these, sleep disturbance is particularly important in the elderly. Strong associations have been observed between depression, fatigue, and poor sleep quality, where circadian rhythm disruption and impaired emotional regulation further intensify fatigue (35). Epidemiological investigations also show that under chronic social stress, elderly cancer patients frequently present with symptom clusters—fatigue, depression, anxiety, insomnia, and attentional deficits—that reinforce one another and contribute to the persistence of CRF (36).

Mechanistically, emotional disorders and fatigue exhibit a bidirectional relationship (37). Two primary pathways have been proposed: (1) neuroimmune dysregulation, including HPA axis disruption leading to loss of cortisol rhythmicity and weakened vagal anti-inflammatory signaling; and (2) maladaptive behaviors, where low self-efficacy reduces activity participation and accelerates muscle metabolic decline (38). Importantly, the “fatigue-depression-insomnia” symptom cluster is highly prevalent in the elderly, while greater psychological resilience and stronger social support can mitigate CRF severity (39).

These findings highlight the need for integrative interventions. Clinical strategies should combine cognitive behavioral therapy to correct catastrophic thinking, family-based support to strengthen coping resources, and resilience training to disrupt the vicious cycle between psychological stress and physiological fatigue.

### 2.3 Cancer- and treatment-induced mechanisms

The physiological and psychological characteristics of elderly patients shape the development of CRF and are directly influenced by the tumor itself and its treatment modalities. Tumor tissues can disrupt nutrient metabolism, induce systemic inflammation, and cause tissue damage, ultimately leading to metabolic imbalance and energy deficits. For instance, patients with esophageal cancer often experience reduced oral intake due to swallowing difficulties, resulting in secondary malnutrition that aggravates fatigue (40).

Cancer treatments—including chemotherapy, radiotherapy, and corticosteroid therapy—are major triggers of CRF exacerbation in older adults. Chemotherapeutic agents, while cytotoxic to tumor cells, also impair hematopoietic function, damage gastrointestinal epithelium, and disrupt neural integrity, thereby intensifying fatigue and weakness. Clinical data demonstrate that CRF symptoms significantly increase during chemotherapy, and advanced age is an independent predictor of persistent fatigue (41).

Treatment-induced mitochondrial dysfunction has been identified as a critical driver of peripheral fatigue. In prostate cancer patients undergoing radiotherapy, decreased activity of mitochondrial respiratory complexes I and IV was negatively correlated with fatigue severity, suggesting that impaired energy metabolism contributes to CRF (42). Furthermore, the combination of androgen deprivation therapy and radiotherapy has been shown to suppress mitochondrial function, induce muscle energy deficits, and correlate with moderate-to-severe CRF (43). These findings underscore mitochondrial dysfunction as a key molecular mechanism underlying treatment-related fatigue.

Given age-associated declines in muscle strength, cardiopulmonary endurance, and immune regulation, elderly patients exhibit heightened vulnerability to treatment-related toxicities compared to younger individuals. This “treatment stress susceptibility” is a distinctive clinical feature of CRF in the elderly.

## 3 Mechanistic overview of VD in the intervention of CRF

**Figure 2:**
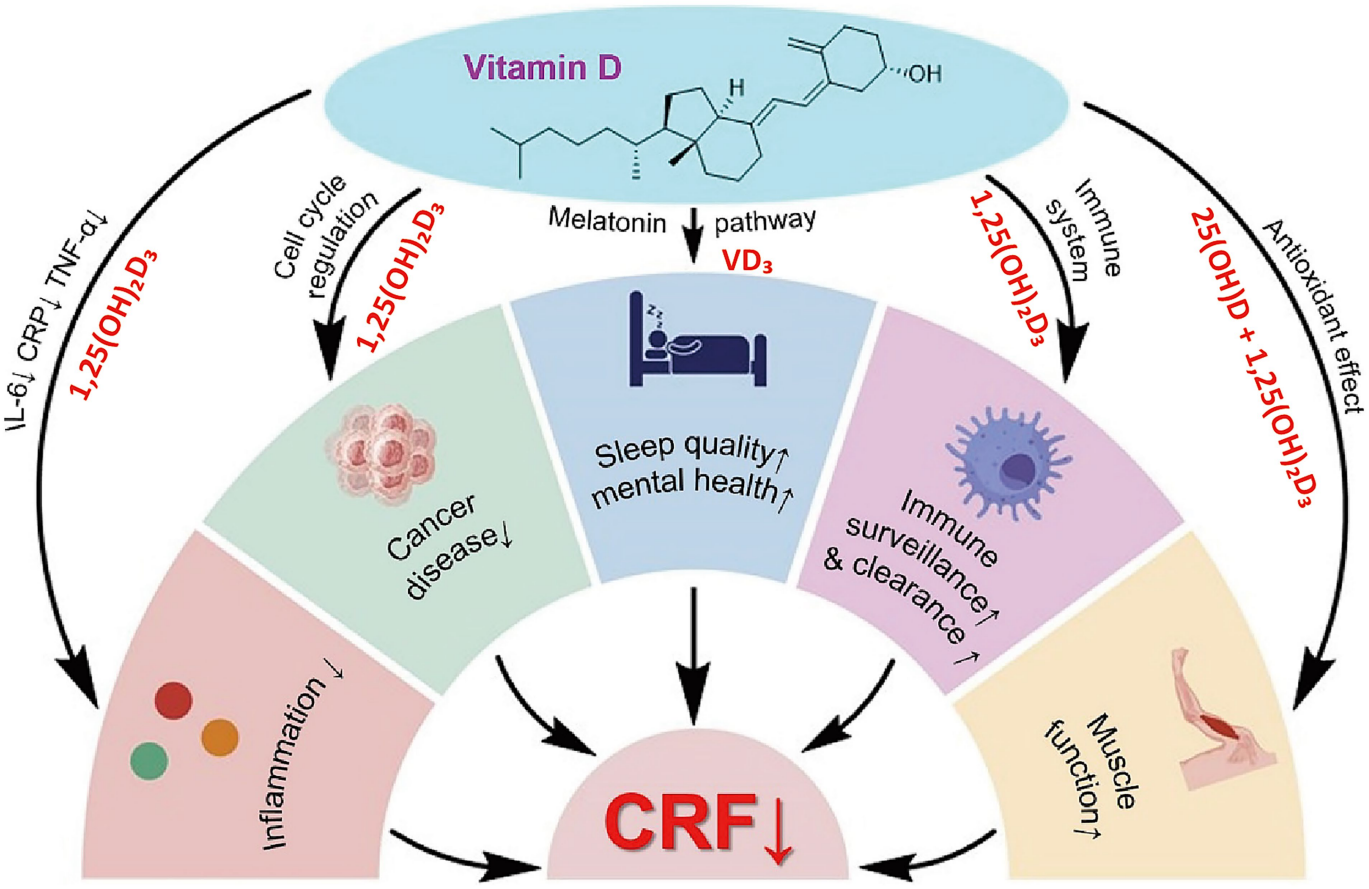
Multimodal integration model of VD in alleviating elderly CRF.

Recent oncology research has extended the role of VD beyond bone metabolism to include immunomodulation, inflammation control, muscle protection, mood regulation, and anti-tumor activity. Evidence indicates that VD deficiency, common in elderly cancer patients, may contribute to or aggravate CRF. This chapter reviews five major mechanisms linking VD to CRF: immunoregulation, cytokine modulation, skeletal muscle preservation, psychological improvement, and anti-tumor pathways. It also discusses the feasibility of VD supplementation as a nutritional intervention in this population (Figure 2).

### 3.1 Immunomodulation

VD is a key regulator of both innate and adaptive immunity. Its active form acts through the vitamin D receptor (VDR), which is expressed in multiple immune cell types, including macrophages, monocytes, dendritic cells, T cells, and B cells (44, 45). Beyond controlling inflammatory responses, VD also contributes to anti-tumor immune surveillance. Studies have shown that VD and its analogs, such as alfacalcidol and paricalcitol, can modulate multiple signaling pathways to promote autophagy and apoptosis in tumor cells, thereby slowing cancer progression (46, 47). Furthermore, VDR is widely distributed across human tissues, and its expression has been closely linked to the occurrence and progression of various malignancies.

In elderly cancer patients, immune function is markedly impaired by aging, disease burden, and treatment-related factors, leading to weakened anti-tumor capacity and elevated systemic inflammation, which in turn exacerbate fatigue (48). Under these conditions, VD supplementation may help restore immune competence, improve tolerance to treatment-related side effects, and alleviate CRF by reducing immune dysregulation-associated fatigue.

### 3.2 Inflammation suppression

VD plays a critical role in regulating inflammation by suppressing pro-inflammatory cytokines and promoting anti-inflammatory mediators, thereby maintaining immune homeostasis. In antigen-presenting cells, VD upregulates IL-10 while downregulating IL-6 and TNF-*α*, thereby limiting the inflammatory cascade (49). A bidirectional Mendelian randomization study revealed a significant negative correlation between serum 25(OH)D levels and CRP concentrations (50). A prospective study in 1,267 pancreatic cancer patients further showed that VD deficiency was associated with elevated IL-6 and CRP levels and reduced overall survival (51), suggesting that VD deficiency may exacerbate inflammation and contribute to the severity of CRF.

A systematic review and meta-analysis of randomized controlled trials reported that VD supplementation significantly reduced CRP and IL-6, with some studies also indicating a decline in TNF-*α* (52), supporting its therapeutic potential in CRF, particularly in elderly patients with chronic low-grade inflammation. Emerging evidence also suggests that diet-induced microbial metabolites may exert their effects through VD signaling pathways to modulate immunity and slow inflammation-driven tumor progression (53). In summary, VD-mediated inflammation control provides a strong theoretical basis and clinical potential for improving CRF in elderly cancer patients.

### 3.3 Protection of skeletal muscle function

VD plays a critical role not only in calcium and phosphorus metabolism but also in regulating skeletal muscle, an area that has garnered increasing attention. Acting through the VDR, VD maintains intracellular calcium balance, mitochondrial function, and antioxidant defense in muscle cells (54). In elderly cancer patients, skeletal degeneration and loss of muscle mass manifest as frailty and heightened peripheral fatigue. Evidence suggests that VD promotes calcium absorption and utilization, supports bone remodeling and fracture healing, and indirectly contributes to physical function (55). Animal studies further confirm that VD deficiency impairs mitochondrial respiratory efficiency and exacerbates muscle fatigue (56).

Clinical research supports these findings. In a study of elderly individuals aged 64–96, VD supplementation improved muscle function, enhanced antioxidant capacity, and delayed fatigue onset, with the greatest benefit observed in those with limited physical activity (57). Together, these results suggest that VD protects skeletal muscle by enhancing mitochondrial metabolism, reducing oxidative stress, and modulating calcium signaling, thereby alleviating peripheral fatigue and improving physical performance and quality of life in elderly cancer patients.

### 3.4 Emotional regulation and sleep-enhancing effects

Psychological disturbances and sleep dysregulation are major amplifiers of CRF in the elderly. Anxiety and depression intensify fatigue, erode dignity, increase dependence, and accelerate disease progression (58–60). V regulates the CNS by upregulating neurotrophic and antioxidant genes and suppressing NF-κB-mediated inflammation, lowering IL-6 and TNF-*α* (61). In tumor-bearing mice, 1,25(OH)_2_D_3_ reduced prefrontal IL-6 by 52%, increased SOD by 38%, and improved exploratory behavior (62). VD also supports melatonin synthesis via the TPH-serotonin-N-acetyltransferase cascade. In lung cancer patients with sleep disturbance, each 5 ng/mL drop in serum 25(OH)D was linked to an 18% reduction in nocturnal melatonin peak (*p* < 0.01) and a 23-min increase in sleep latency (63); TPH-2-dependent serotonin-melatonin conversion has also been implicated (64). Higher VD status is associated with reduced depression incidence and severity (65).

Clinical evidence further confirms these links. Depression independently predicts CRF (OR = 2.23; 95% CI: 1.70–2.93, *p <* 0.001) (12). VD deficiency increases sleep disorder risk (OR = 1.50; 95% CI: 1.31–1.72, *p <* 0.001) (66). Overall, VD may mitigate CRF by suppressing NF-κB-driven neuroinflammation, enhancing antioxidant defense, and stabilizing serotonin-melatonin pathways. Most data derive from basic and observational studies; rigorous trials are needed to establish dosage, mechanisms, and timing in elderly patients with cancer.

### 3.5 Anti-tumor mechanisms

VD exhibits multidimensional regulatory functions in anti-tumor mechanisms. Epidemiological studies link low VD levels to a higher risk of colorectal, breast, lung, and prostate cancers (67–71). At the molecular level, VD binds to the VDR and regulates pathways controlling growth, differentiation, and apoptosis. It inhibits tumor proliferation by modulating cyclin-dependent kinases and arresting the cell cycle, while promoting apoptosis through the induction of pro-apoptotic factors such as Bax and p21 (72, 73). VD also reshapes the tumor microenvironment by influencing glucose, glutamine, and lipid metabolism, thereby enhancing metabolic resilience and reducing tumor viability through antioxidant mechanisms (74). In elderly cancer patients, these effects may slow disease progression, reduce wasting, and indirectly alleviate CRF by decreasing tumor burden and inflammation.

In summary, VD exerts integrative effects on CRF through multiple targets (Figure 2). Immunologically, it regulates immune cells to maintain homeostasis. In inflammation control, it downregulates IL-6 and TNF-*α*. In skeletal muscle, it supports mitochondrial function and reduces fatigue. In psychological regulation, it exhibits antidepressant and sleep-enhancing benefits. In anti-tumor pathways, it restrains proliferation and modulates metabolism, thereby lowering the disease burden. Although large-scale trials specifically addressing VD in CRF remain limited, current evidence supports its potential as a safe, multifaceted intervention. Future research should refine dosage, timing, and duration to enable precise application across nutritional, immunological, and psychological domains.

## 4 Clinical characteristics and intervention challenges of CRF in elderly cancer patients

CRFin elderly cancer patients presents significant clinical management challenges, with under-recognition being the foremost barrier. A survey of 2,508 cancer patients showed that more than 58% were unaware of fatigue as a treatment-related complication, and the information deficit was particularly pronounced in those aged ≥ 70 years (65% vs. 52% in patients under 60) (75). This misconception often leads elderly individuals to regard fatigue as “normal aging,” thereby delaying intervention. Moreover, CRF in the elderly displays greater heterogeneity. In addition to physical weakness, patients frequently exhibit cognitive slowing, poor concentration, emotional blunting, and sleep disturbances (76). Compared with younger patients, whose fatigue is largely physical, older adults more often present with “biopsychosocial fatigue,” complicating recognition and assessment (77). Intervention adherence further compounds these challenges. Cognitive decline, comorbidities, polypharmacy, and reduced physical capacity often hinder sustained participation in exercise or psychological therapies (78, 79). Limited family support, communication barriers, and informational burdens exacerbate systemic adherence issues (80).

**Figure 3:**
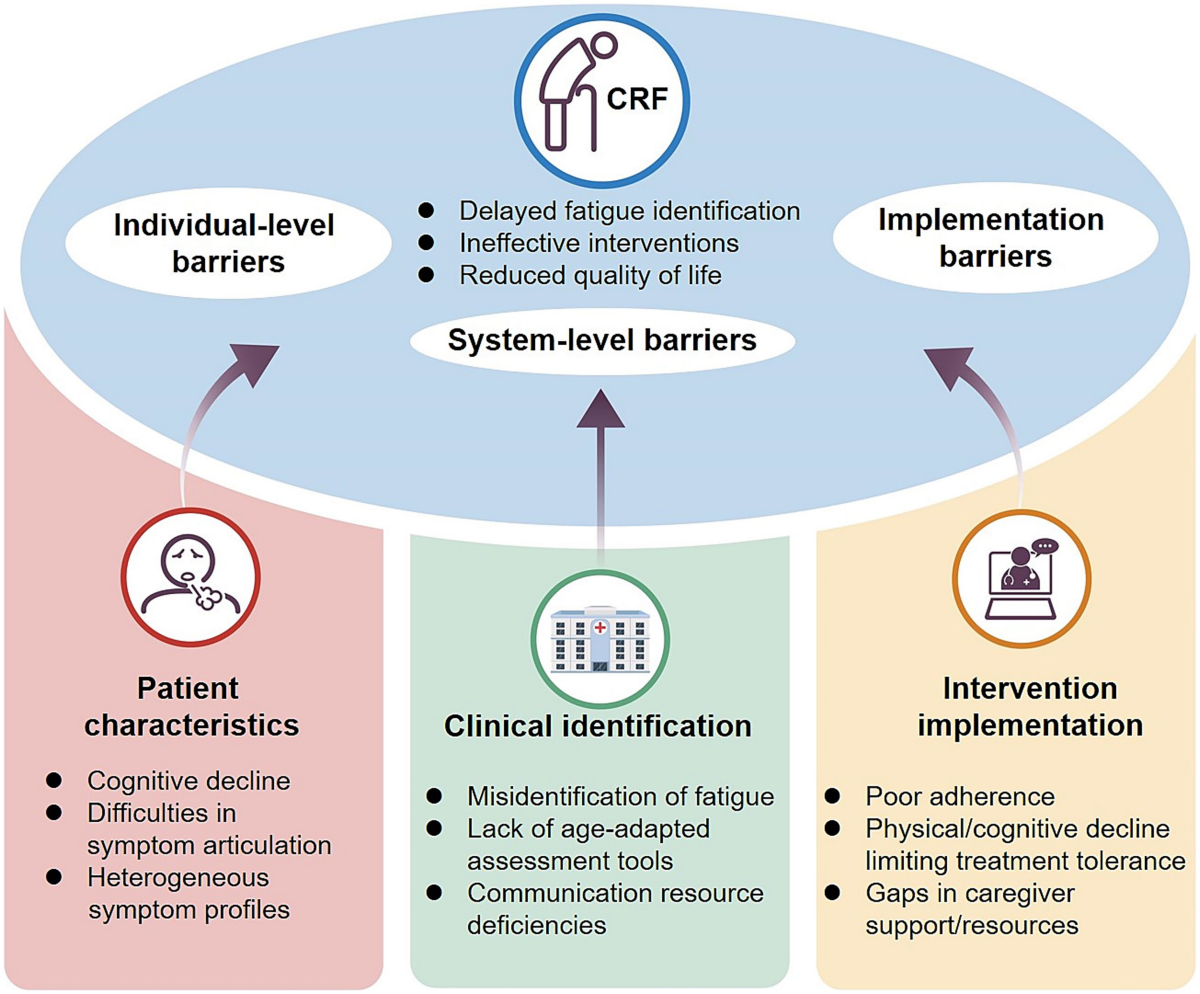
Pathway map of clinical identification and intervention barriers in elderly CRF.

Elderly CRF should therefore be recognized as a multidimensional syndrome encompassing biological, psychological, and social domains (Figure 3). Its heterogeneity, diagnostic difficulties, and limited suitability of current interventions distinguish it from “normal aging fatigue” and underscore the need to consider it as an independent clinical entity. Future efforts should focus on developing dedicated screening tools and comprehensive intervention models tailored for older adults, integrating etiology, symptom profiling, and adherence strategies to improve quality of life and treatment outcomes.

## 5 Intervention strategies for CRF: current status and future directions

**Figure 4:**
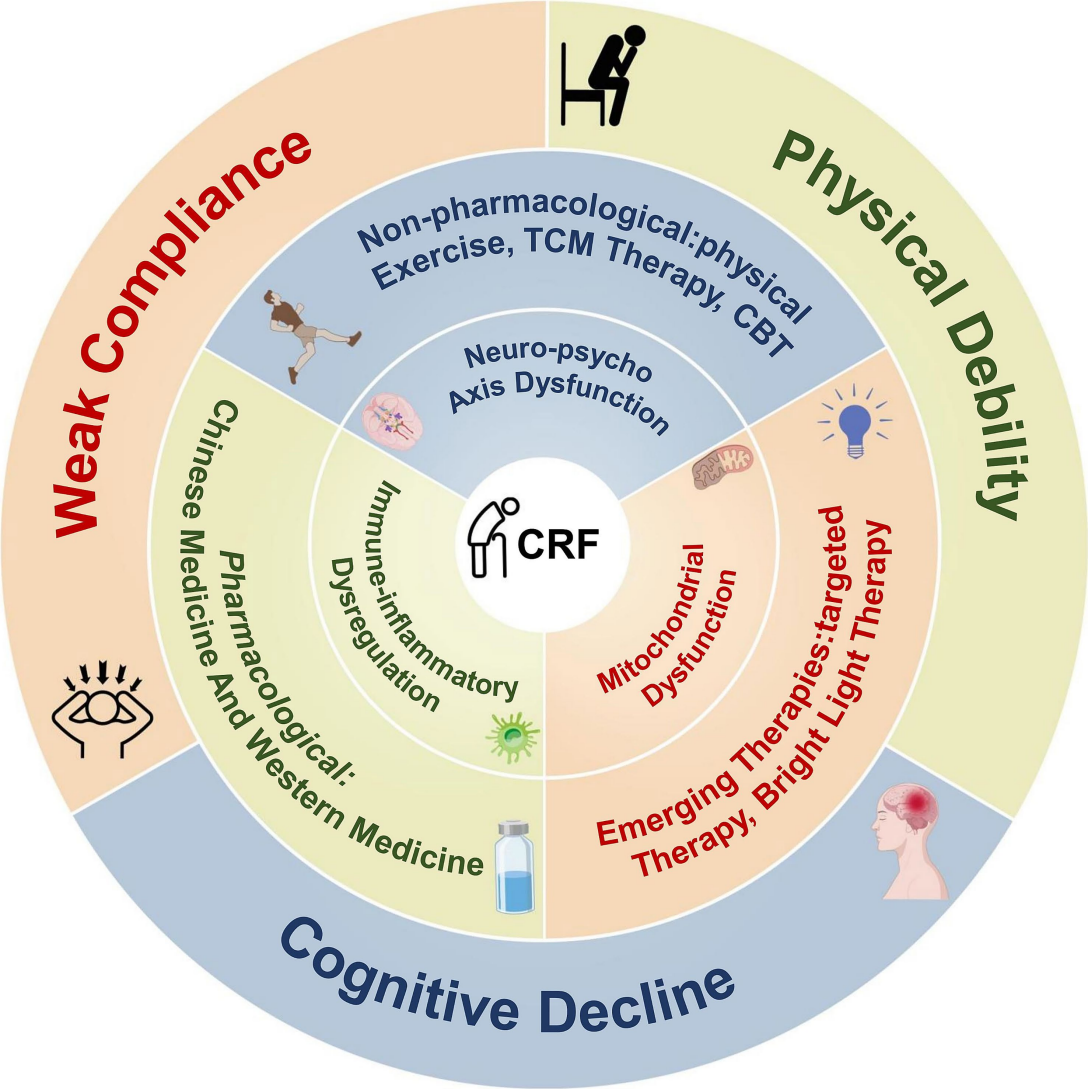
Intervention landscape for CRF: integration of multidimensional mechanisms and therapeutic pathways.

CRF is one of the most common and debilitating symptoms during cancer and its treatment, substantially reducing quality of life and treatment compliance. Intervention strategies have expanded considerably in recent years, spanning pharmacological treatments, non-pharmacological approaches, and emerging biologically based methods. Nevertheless, inconsistent efficacy and the lack of individualized application remain major barriers to clinical translation. This section reviews three primary categories of interventions, outlining their theoretical underpinnings, current evidence, and future research trends (Figure 4).

### 5.1 Pharmacological interventions

Pharmacological therapy is the most commonly employed first-line intervention for CR and can be broadly categorized into two main types: traditional Chinese medicine (TCM)-based compound formulations and conventional Western pharmacological agents. While certain drugs have demonstrated potential in alleviating CRF symptoms, their overall efficacy remains uncertain, and concerns regarding side effects and limited applicability persist. In elderly patients, where individual variability is pronounced and comorbidities are frequent, careful evaluation of the risk–benefit balance of pharmacological interventions is particularly critical.

#### 5.1.1 TCM

In TCM, CRF is often classified under the category of “deficiency syndromes,” commonly attributed to patterns such as “qi and blood deficiency,” “liver and kidney insufficiency,” and “spleen and stomach disharmony.” Treatment typically emphasizes tonifying qi and blood, nourishing the liver and kidneys, and strengthening the spleen and middle burner. Frequently used formulations include Chuanhuang Oral Liquid, Renshen Yangrong Decoction, and Compound Ejiao Syrup. Buzhong Yiqi Decoction (BYD), a classical formula originally used for spleen deficiency and qi prolapse, has recently been shown to improve immune regulation and energy metabolism. A study by Hu et al. demonstrated that BYD alleviated CRF symptoms in cervical cancer patients by promoting protein synthesis and enhancing immune responses (81). Additionally, a systematic review and meta-analysis by Li et al., which included 1,777 patients, found that Chinese herbal medicine interventions significantly improved fatigue symptoms (OR = 2.81; 95% CI: 1.78–4.41; *p <* 0.001) (82).

Although TCM has gained attention for its multi-target and holistic regulation properties, its clinical application is challenged by the complexity of its components, heterogeneity in drug metabolism, and issues related to quality control and herb compatibility. These concerns are particularly relevant in elderly patients with multiple comorbidities, where there is an increased risk of adverse reactions and drug interactions. Therefore, the clinical use of TCM in elderly CRF patients should proceed cautiously, based on standardized research and thorough assessments of individual tolerance.

#### 5.1.2 Western pharmacological treatment

In Western medicine, various agents have been tested for CRF, including antidepressants (paroxetine, bupropion), central stimulants (methylphenidate, modafinil), and corticosteroids (dexamethasone). Most studies show only moderate benefit in selected patients, with inconsistent evidence and no consensus in guidelines (83–87). In a six-week randomized trial, bupropion 150 mg/day reduced fatigue scores compared to placebo (4 ± 0.9 vs. 4.9 ± 0.7, *p =* 0.006), with effects emerging after four weeks and paralleling improvements in function and mood (85). In older patients, however, altered pharmacokinetics, polypharmacy, and poor tolerance necessitate cautious use, with interventions best targeted to cases of central or symptom-dominant fatigue.

### 5.2 Non-pharmacological interventions

With advances in understanding CRF mechanisms, an increasing number of non-pharmacological interventions can improve CRF among elderly cancer patients, offering advantages such as better safety, higher adherence, and fewer side effects. Current mainstream non-pharmacological strategies include exercise training, TCM techniques (e.g., acupuncture, moxibustion), and cognitive behavioral therapy (CBT). Some studies have also explored combining these methods into multimodal integrated intervention programs.

#### 5.2.1 Exercise

Exercise is widely recognized as the most effective and well-supported non-pharmacological intervention for CRF, particularly in elderly patients. Resistance training promotes neuromuscular reinnervation, preserves muscle mass, and enhances contractile function, thereby improving physical performance and self-care ability (88). A multicenter RCT demonstrated that individualized rehabilitation significantly improved physical capacity, muscle function, and reduced FACT-F scores (81.13 ± 14.81 vs. 107.09 ± 28.31, *p* < 0.05) (89). Systematic reviews and meta-analyses indicate that moderate-to-high intensity exercise has stronger effects on CRF, although adherence remains limited in older adults (90). Gentler exercise forms, such as Baduanjin, Tai Chi, and walking, are increasingly emphasized. In one RCT, Baduanjin combined with nutritional support significantly reduced fatigue in elderly lung cancer patients (4.54 ± 1.19 vs. 3.75 ± 0.99, *p* < 0.05) (91). A network meta-analysis found that Tai Chi, walking, and resistance training were all beneficial for CRF (92). Remote exercise programs have also shown feasibility in patients with limited mobility (93).

Elderly patients generally prefer mild activities such as walking, Tai Chi, or Baduanjin. In prostate cancer, Baduanjin has been shown to reduce pain, improve muscle tone, modulate immune-inflammatory responses, and enhance neural reactivity (94). In breast cancer patients receiving radiotherapy, progressive home-based walking was not superior to usual care, but fatigue reduction correlated positively with increased walking (95). A meta-analysis of 113 RCTs involving 11,525 patients showed that psychological interventions had a significant effect on CRF (WES = 0.27, *p* < 0.001), comparable to exercise (WES = 0.30), and superior to pharmacological treatments (WES = 0.09, *p* = 0.05) (96). In summary, exercise provides clear physiological and psychological benefits for elderly CRF patients, but its effectiveness depends on individualized adaptation, tolerance, and adherence, and may be enhanced by integration with multimodal approaches.

#### 5.2.2 TCM therapies

TCM holds a unique role in the management of CRF, particularly among elderly patients in East Asian Confucian cultural contexts. These populations often recognize TCM etiological concepts (e.g., imbalance of qi, blood, yin, and yang), show high adherence to non-pharmacological therapies such as acupuncture and moxibustion, and value the holistic care model, making TCM a preferred strategy for multidimensional management. Common interventions include acupoint massage, acupuncture, and moxibustion. Acupoint massage is valued for its safety and accessibility, while acupuncture targets core pathological processes through multisystem regulation (97, 98). Pressing specific acupoints twice daily for 3 min over 4 weeks significantly reduced fatigue scores in elderly cancer patients (99). Acupuncture has also been shown to improve fatigue in breast cancer patients (100).

Infrared laser moxibustion has attracted increasing attention. A randomized controlled trial in breast cancer survivors reported that 18 weeks of intervention led to significant reductions in BFI fatigue scores, with differences of −0.9 points at week 6 (95% CI: 0.3–1.6, *p* = 0.007) and −1.1 points at week 18 (95% CI: 0.4–1.8, *p* = 0.002), suggesting delayed onset but sustained benefit (101). A multicenter RCT confirmed its effectiveness in alleviating CRF after chemoradiotherapy, with high adherence and minimal side effects (102). Novel 3.95 μm infrared moxibustion further improved fatigue, sleep quality, and autonomic function, likely through combined modulation of the brain-gut axis and immune-inflammatory pathways (103). Overall, TCM demonstrates multidimensional benefits with strong patient acceptance, especially in acupoint massage and moxibustion supported by RCTs. Future studies should clarify its synergistic role in multimodal treatment frameworks and validate mechanisms across neuroimmune and psychosocial pathways.

#### 5.2.3 CBT

CBT is a structured psychological intervention that alleviates emotional distress and maladaptive behaviors through cognitive restructuring, emotional regulation, and behavioral training. In elderly cancer patients, CRF is often accompanied by anxiety, depression, social withdrawal, and functional decline. A meta-analysis of 10 RCTs confirmed the robust efficacy of CBT in reducing fatigue (standardized mean difference (SMD) = −2.50, 95% CI: −3.43 to −1.56, *p* < 0.001) (104). Clinical studies also report high adherence and satisfaction among older patients, with evidence supporting the use of CBT for managing symptom clusters. For example, CBT for insomnia improved sleep efficiency and alleviated CRF (105, 106). In contrast, a CBT-TTF model tailored to targeted therapy-related fatigue effectively reduced symptoms through sleep hygiene and activity planning (107).

The flexibility of CBT formats enhances its adaptability for elderly patients. Structured activity scheduling strengthens self-efficacy and long-term adherence (108), and Acceptance and Commitment Therapy (ACT), based on CBT principles, emphasizes caregiver involvement, value-driven behaviors, and resilience training, showing potential in advanced cancer populations (109). Overall, CBT demonstrates significant effectiveness in CRF management by improving multidimensional symptoms, supporting adherence, and fostering psychological recovery. Future directions should emphasize personalized design, digital delivery (e.g., remote CBT), and multidisciplinary integration for elderly patients with comorbidities.

### 5.3 Emerging interventions (bright light therapy (BLT), mitochondrial-targeted approaches)

As research into the mechanisms of CRF deepens, several emerging therapies have been introduced and are currently being evaluated in clinical trials. These interventions target key pathological processes, including mitochondrial dysfunction, disruption of the circadian rhythm, and neuroendocrine abnormalities. Characterized by multi-target effects, low side-effect profiles, and potentially high adherence, these strategies are not yet widely adopted but have shown promising adaptability in elderly patients with cancer.

#### 5.3.1 BLT: circadian rhythm and neuroendocrine regulation

BLT is a non-pharmacological intervention that acts on the suprachiasmatic nucleus to regulate circadian rhythms, thereby influencing the secretion of melatonin and cortisol, and improving sleep, mood, and neural function (110). Studies have shown that BLT enhances HPA axis function and reduces fatigue in elderly patients with depression (111, 112), supporting its theoretical basis for CRF management.

Two systematic meta-analyses further validated its efficacy. One included 13 trials (intervention periods ranging from 1–12 weeks, light intensity 417.9–12,000 lux) and reported a moderate effect on CRF (SMD = 0.45, *p* = 0.007) (113). Another, more recent meta-analysis including 12 trials, found a significant reduction in CRF (SMD = −0.92, *p* < 0.0001), recommending protocols of ≥ 10,000 lux for at least 4 weeks (114). With its non-invasive nature, minimal side effects, and low cost, BLT demonstrates strong suitability for elderly patients; however, standardized parameters for geriatric oncology populations have not yet been established.

Emerging therapies, such as BLT, offer multiple advantages, including multi-target mechanisms, a non-invasive safety profile, good tolerability, and high patient acceptance. Whether by modulating mitochondrial function or reshaping neurohormonal rhythms, these approaches broaden CRF management strategies. Future studies should clarify geriatric-specific efficacy and parameters, integrate BLT with conventional interventions (e.g., exercise, CBT), and strengthen mechanistic validation to shift CRF management from symptomatic relief to mechanistic regulation.

#### 5.3.2 Targeted therapy: mitochondrial function regulation

Targeted therapies have traditionally been applied to tumor control, but mitochondrial dysfunction is now recognized as a key mechanism of CRF. Elevated circulating mitochondrial DNA (mtDNA) levels correlate with fatigue severity in cancer patients, suggesting that energy imbalance mediated by mitochondrial impairment contributes to CRF (115).

Among potential interventions, coenzyme Q10 (CoQ10) has shown promising results. In a randomized trial of 57 middle-aged and elderly breast cancer patients, daily supplementation with 100 mg CoQ10 for three weeks significantly reduced fatigue compared with placebo: maximum fatigue decreased by 43.1% versus 2.9%, overall fatigue by 30.1% versus 6.3%, and current fatigue by 53.8% versus 6.3% (all *p* < 0.001) (116). Although still in the exploratory phase, mitochondrial-targeted strategies may represent a novel avenue for CRF management, warranting larger and longer-term trials to evaluate safety and patient-specific responses, particularly in elderly populations with multiple comorbidities.

## 6 Review and analysis of evidence on the role of VD in alleviating CRF

CRF is defined as a persistent, multidimensional subjective sense of tiredness caused by cancer itself or its treatments (e.g., chemotherapy, radiotherapy, targeted therapy). It involves physical, emotional, and cognitive domains, is disproportionate to recent activity, and cannot be relieved by rest (4, 117). Diagnosis requires integrating standardized patient-reported scales, objective inflammatory and metabolic biomarkers, and excluding comorbidities (118, 119).

VD, as a precursor to corticosteroids with immunomodulatory, anti-inflammatory, neuroprotective, and muscle metabolism-regulating properties, has garnered increasing attention for its potential role in the intervention of CRF. Although a unified treatment consensus has not yet been established, multiple clinical studies have investigated the relationship between VD levels and CRF, including observational studies, RCTs, and Mendelian randomization analyses. This section systematically reviews and analyzes the current body of evidence, considering study design, intervention dosage, follow-up duration, and outcome measures (Table 1). VD, with its immunomodulatory, anti-inflammatory, neuroprotective, and muscle metabolic effects, has emerged as a potential intervention for CRF. Mechanistically, VD enhances mitochondrial respiratory efficiency, promotes fatty acid oxidation, downregulates pro-inflammatory cytokines such as IL-6 and TNF-*α*, and improves muscle function and immune surveillance (120–123). Clinical and non-cancer studies further suggest that VD supplementation may reduce fatigue, slow disability progression, and modulate neuroimmune function (124, 125).

**Table 1:** Summary of studies on vitamin D interventions for cancer-related fatigue (CRF).

Study author	Study type	Sample size	VD intervention method	Follow-up duration	Fatigue outcomes
Custódio al. (38)	Observational	89	−75 nmol/L	24 months	Vitamin D deficiency increases aromatase inhibitor toxicity and promotes fatigue
Koole et al. (126)	Cohort study	261	Serum VD levels	2 years	Increased VD associated with significantly reduced fatigue scores (EORTC-QLQ-C30)
Schöttker et al. (8)	RCT	456	High dose + 2000 IU/day maintenance	12 weeks	Trend toward fatigue improvement; supports tertiary prevention
Nowak et al. (127)	RCT	120	Single dose of 100,000 IU	4 weeks	Significant reduction in fatigue scores (*p* = 0.01)
Khan et al. (128)	RCT	60	Standard VD supplementation	5 weeks	No significant difference (*p* = 0.15)
Havdahl et al. (129)	Mendelian randomization	33,607	Genetically predicted VD levels	4 years	No causal association between VD levels and fatigue supported

Clinical evidence supports these findings. Observational studies show that VD deficiency is associated with exacerbated drug toxicities and increased fatigue risk (38), while higher VD concentrations correlate linearly with lower fatigue scores (126). Interventional studies report that high-dose short-term supplementation (single dose of 100,000 IU) significantly improved fatigue (*p* = 0.01) (127), and intensified regimens (20,000–40,000 IU/day loading followed by 2,000 IU/day maintenance for 12 weeks) demonstrated an improvement trend (8). However, smaller trials have not observed significant effects (128), highlighting the influence of dose, duration, and population-specific factors. At the genetic level, a Mendelian randomization analysis (*n* = 33,607) found no causal association between serum VD levels and CRF risk (129), underscoring heterogeneity across studies.

**Figure 5:**
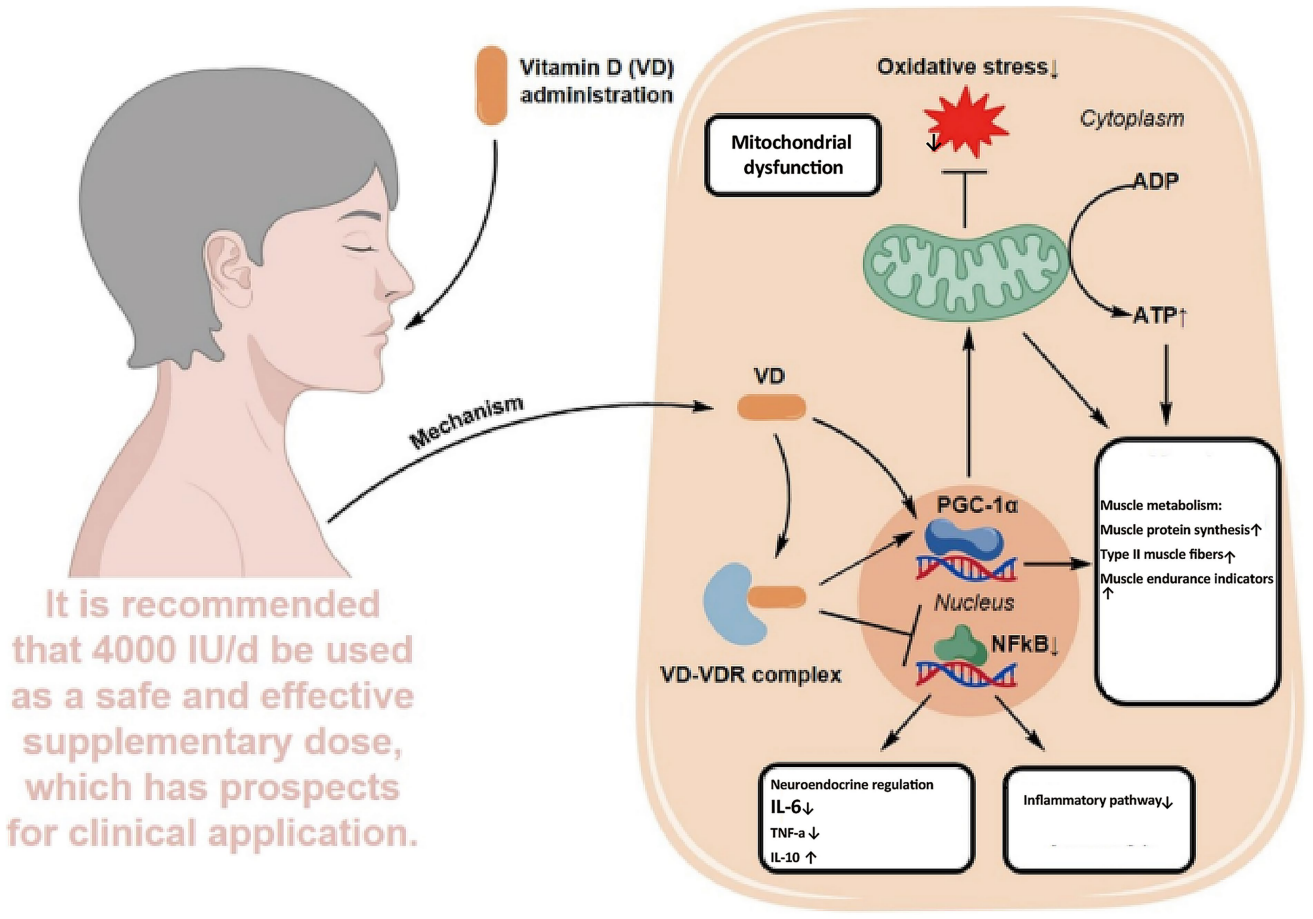
Mechanistic schema of VD supplementation in improving CRF.

Overall, VD supplementation may benefit CRF management, particularly in patients with VD deficiency and potentially in the elderly, who are more vulnerable to CRF due to physiological decline. Yet, dedicated subgroup studies in older populations remain lacking. Future large-scale, stratified RCTs, mechanistic investigations, and real-world evidence are needed to clarify dose–response relationships, determine optimal supplementation regimens, and establish the safety and applicability of VD interventions in elderly cancer survivors (Figure 5).

## 7 General discussion: mechanistic integration, clinical value, and practical challenges

CRF is a multidimensional syndrome triggered by cancer and its treatments, involving immune-inflammatory activation, neuroendocrine dysregulation, mitochondrial impairment, skeletal muscle decline, and psychosocial maladaptation. In elderly patients, reduced physiological reserve, immunosenescence, and diminished neuroplasticity amplify these mechanisms, making symptoms more persistent and less responsive to interventions. Thus, mechanistic understanding requires a systems-level perspective that incorporates aging-specific processes.

VD, through its anti-inflammatory, immunoregulatory, neuromodulatory, and muscle metabolic effects, has been proposed as a potential modulator of CRF. Observational studies show VD deficiency correlates with higher fatigue, while some randomized trials report improvements after high-dose or short-term supplementation, particularly in breast and colorectal cancer patients. However, heterogeneous study designs, variable dosing strategies, and inconsistent measures of fatigue limit cross-study comparability. By contrast, non-pharmacological interventions such as exercise, CBT, acupuncture, and bright light therapy demonstrate better acceptability in older adults and appear especially promising when integrated into multimodal or remote management programs.

Key challenges remain. First, evidence on VD dosing and efficacy in elderly subgroups is lacking, while high-dose regimens may carry metabolic or drug-interaction risks. Second, although exercise and CBT are effective, adherence is often constrained by functional limitations, caregiving responsibilities, and limited digital literacy. Third, the absence of reliable biomarkers to predict treatment response hampers personalized management. Future studies should prioritize stratified randomized controlled trials, multimodal approaches (e.g., VD combined with immunotherapy or resistance exercise), and dynamic biomarker validation (inflammatory mediators, mitochondrial function, VDR polymorphisms), supported by long-term follow-up. Such designs may clarify optimal dosing, timing, and patient selection, advancing CRF management toward evidence-based and individualized care.

## 8 Conclusion and recommendations

CRF is one of the most common and debilitating persistent symptoms in elderly cancer patients, characterized by marked heterogeneity and complex pathophysiology. This review synthesized current evidence on CRF mechanisms, elderly-specific features, the role of VD, and available intervention strategies. VD shows multi-target potential in immunoregulation, inflammation suppression, muscle preservation, and psychological modulation. Observational and interventional studies suggest benefits in selected populations and dosages, yet the overall evidence remains insufficient, particularly for elderly patients. Standardized protocols and individualized dosing require further validation in high-quality, stratified prospective trials.

In clinical practice, VD assessment should be integrated into CRF risk evaluation, with supplementation tailored to nutritional status, comorbidities, treatment stage, and cognitive function. Non-pharmacological interventions, including exercise, CBT, acupuncture, and bright light therapy, should remain first-line strategies to ensure safety and improve management outcomes.

From a research perspective, future work should: (1) incorporate genomic profiling to explore VDR polymorphisms, mitochondrial genes, and inflammatory pathways regulating VD responsiveness, identifying sensitive subgroups for clinical targeting; (2) build large-scale dose–response models across age groups to define safe and effective dosing frameworks for the elderly; (3) advance multimodal approaches by combining VD with anti-inflammatory nutrients, antioxidants, muscle-enhancing agents, and non-pharmacological therapies for synergistic effects; and (4) leverage telemedicine, wearable devices, and digital platforms to enable real-time VD monitoring, fatigue self-reporting, and adaptive interventions.

In summary, precision nutrition with VD should evolve beyond static supplementation toward a data-driven, interdisciplinary model integrated into real-world care. Such an approach may establish VD as a core component of comprehensive, personalized CRF management in elderly cancer survivors.
